# D8/17 Monoclonal Antibody: An Unclear Neuropsychiatric Marker

**DOI:** 10.1155/2005/795343

**Published:** 2005-08-04

**Authors:** Astrid Morer, Odette Viñas, Luisa Lázaro, Jordi Bosch, Josep Toro, Josefina Castro

**Affiliations:** ^1^Department of Child and Adolescent Psychiatry and PsychologyHospital ClínicBarcelonaSpain; ^2^Department of Immunology Hospital ClínicBarcelonaSpain; ^3^Department of MicrobiologyHospital ClínicBarcelonaSpain; ^4^Institut d'Investigació Biomédica August Pi i Sunyer (IDIBAPS)Spain

**Keywords:** D8/17, B lymphocytes, PANDAS, obsessive compulsive disorder, flow cytometry

## Abstract

*Objective:* It has been hypothesized that monoclonal antibody D8/17 identifies a B lymphocyte antigen with expanded expression in patients with rheumatic fever, childhood onset obsessive-compulsive disorder (OCD), Tourette syndrome (TS) or prepubertal anorexia nervosa (AN). Our purpose was to replicate these studies in a Spanish population and to determine whether D8/17 identifies a subgroup of these patients, focusing especially on OCD subjects.

*Method:* D8/17 expression was assessed with double immunofluorescence and flow cytometry using monoclonal immunoglobulin M (IgM) in three groups of patients with diagnoses of OCD (*n = 17*), TS (*n = 5*) and prepubertal AN (*n = 5*), recruited during 2001.

*Results:* In the sample studied the average percentage of B cells expressing D8/17 was 4.8%. The D8/17 positive proportion of B lymphocytes was above 11% in only two out of 17 OCD patients (7.4% of total sample) and in none of the TS or prepubertal AN patients. No statistically significant differences were found in mean percentages of D8/17 between the three groups.

*Conclusions:* In the sample studied the expression of D8/17 in B cells was very low and the great majority of patients were negative for the D8/17 marker. The molecular characterization of D8/17 would be a major step forward in clarifying its implication for these diseases.

## 1. Introduction

Significant progress has been made in the treatment of obsessive-compulsive disorder (OCD) and related illnesses. Nonetheless, the identification of reliable clinical and/or biological markers of homogeneous subgroups would increase our understanding of the pathophysiology of the condition, improve treatment, and possibly even help prevention. The presence of comorbid tics has been shown to be a clinical marker for a subgroup of OCD disorders that differ in how they develop and respond to treatment [1]. In addition to genetic factors, autoimmunity may be involved in these disorders. Sydenham's chorea (SC), a neuropsychiatric syndrome that usually occurs in prepubertal children is an excellent example of a condition that helps explain the relation between OCD or related illnesses, such as Tourette syndrome (TS), and immunological dysfunction. SC may provide a medical model for the underlying causes of childhood onset OCD [2].

PANDAS (Pediatric Autoimmune Neuropsychiatric Disorders Associated with Streptococcal infections) is a concept applied to children presenting a dramatic onset of tics and/or OCD caused or exacerbated by group A beta-Hemolytic streptococcus (GABHS) infections. Diagnostic criteria proposed for PANDAS include the presence of OCD and/or tic disorder, pre-

ISSN 0953-4180/05/$17.00 © 2005 – IOS Press and the authors. All rights reserved pubertal symptom onset, episodic course of symptom severity with dramatic symptom exacerbation, association with GABHS infection, and association with neurological abnormalities [3]. It has been postulated that the PANDAS syndrome results from antistreptococcal immune response antibodies that cross-react with basal ganglia tissue, as has been demonstrated in SC [4]. It has been proposed that the M protein expressed on streptococcal cell walls shares homology with host basal ganglia antigens, and that autoimmune induction may involve a process of molecular mimicry [5].

The identification of a molecular marker for rheumatic fever (RF) began at the end of the 1970s, when a B cell alloantigen, which reacted with B cell from RF patients was isolated [3]. Afterwards, Zabriskie and colleagues produced two monoclonal antibodies which used in combination identified 92% of patients with RF versus 21% of controls [6]. Later, the same group developed a monoclonal antibody which identified a B cell antigen present in 100% of all RF patients studied [7]. This monoclonal antibody was called D8/17 (mAb D8/17). It has also been reported that vulnerability to PANDAS probably involves genetic factors that may be related to the increase in the D8/17 positive B lymphocytes subpopulation. D8/17 has been suggested to be a marker of susceptibility to PANDAS [4, 8]. Although at the beginning, the studies found similar results across different ethnic populations and geographic regions, other researchers with new methodological approaches have shown that the discriminatory ability of D8/17 may be reduced due to antigenic variation in different ethnic groups [9]. Larger studies with improved technology would provide a more definitive conclusion [10].

As the diagnosis of SC is often obtained by exclusion, increased expression of D8/17 has been proposed as a potentially useful indicator for differentiating between SC and other forms of chorea. More recently, the possibility of an immune-mediated pathogenesis of OCD/TS has generated interest in the potential of monoclonal antibody D8/17 to identify patients with, or at risk of streptococcus-precipitated neuropsychiatric disorders. Of the studies published to date, increased rates of binding of this monoclonal antibody to B cells have been reported in patients meeting criteria for PANDAS [4] childhood-onset OCD/TS [8], TS [11], AN [12,13] and autism [14].

The hypothetical diagnostic value of this antibody and its relationship to the pathophysiology of psychiatric disorders has yet to be established. Two recent studies have yielded less than satisfactory results. One, a large community study of 240 children with 2–5 year follow-up concluded that D8/17 was unable to provide support as a marker of susceptibility in tics or OCD [15]. The other, a study of 26 SC subjects, 42 OCD or tic disorders (PANDAS subgroups) and 19 healthy controls found the sensitivity of the D8/17 assay to be unacceptable during the period of observation [16]. Although some studies show that D8/17 binding seems to be specifically increased in patients with RF when compared with other rheumatic illnesses, assessment in several neuropsychiatric conditions is needed to confirm its diagnostic specificity.

The purpose of this study was to evaluate the diagnostic value of the D8/17 antibody in a Spanish population. A positive result would support the immunological hypothesis for a subgroup of OCD, TS and prepubertal AN patients.

## 2. Materials and methods

### *2.1. Subjects*

Twenty-seven patients (14 males, 13 females) aged 9 to 16 years old were included. Written informed consent from the parents was obtained for all subjects. All had primary DSM-IV diagnoses of either childhood onset OCD ($n = 17$) 62.9%, TS ($n = 5$) 18.5%, or prepubertal AN ($n = 5$) 18.5%. The patients were recruited from the outpatient clinic of the Department of Child and Adolescent Psychiatry and Psychology at the Hospital Clinic, Barcelona, Spain. To assess OCD severity the Children Yale Brown Obsessive Compulsive Scale [17] -a modified version of the Yale Brown Obsessive Compulsive Scale [18,19]- was used, and the presence of tics was evaluated with the Yale Global Tic Severity Scale [20]. To assess the AN clinical severity the Eating Attitudes Test [21] was used.

All the patients in the OCD and TS groups were receiving drug therapy (serotonin uptake inhibitors and risperidone respectively). None with prepubertal AN was medicated. Each patient and his/her family were questioned carefully about the time relation between onset of neuropsychiatric symptoms after streptococcal infection or streptococcal-related symptom exacerbations, and personal and family history of immunological disease.

### 2.2. Immunology

D8/17 antibody was a generous gift from Dr. J. Zabriskie, Rockefeller University, New York, NY.

Peripheral blood was obtained by venipuncture and collected in vacutainer tubes containing ACD anticoagulant, during the same week that psychiatric assesment was done. Peripheral blood immunofluorescence-staining was performed as previously described [22] in fresh whole blood.

Briefly, the immuno-staining was performed by adding 5 microl of CD 19-PE and 5 microl of CD 3-PerCP, 50 microl of an irrelevant IgM monoclonal antibody as isotype control, CD8 (tube 1), or 50 microl of the D8/17 (diluted 1:50) specific monoclonal antibody (tube 2), to 50 microl of whole blood. After 1 hour of incubation at 4°C, the blood cells were washed twice with 2 ml of phosphate-buffered saline supplemented with 1°/oo Na N3 and 2% of FCS (fetal calf serum). The pellets were then incubated with the appropriate dilution of an FITC conjugated goat antimouse IgM. After 30 minutes of incubation at 4°C two more identical washes were performed, and the cells were analyzed in the flow cytometer within 4 hours. CD 19-PE was used as a marker for the total B subpopulation, CD-3-PerCP was used to exclude T lymphocytes from the total lymphocyte analysis and the fluorescein isothiocyanate conjugated goat antimouse IgM was able to detect binding of the D8/17 specific monoclonal antibody. After incubation, the cells were lysed with lysing solution for 10 minutes, centrifuged and washed.

The immunofluorescence was analysed using a FACScan flow cytometer and the CellQuest software. The proper levels of amplification for the cytometer photo detectors (FL1, FL2, FSC, SSC) and the appropriate compensation set-up were established in order to obtain the cytometer calibration set-up for the experimental conditions. This same calibration was used for all the determinations of the present study. A thousand events were obtained that fulfilled the requisites of two gates established for each experiment. One gate selected lymphocytes according to the FSC and SSC characteristics and the other gate was used to select the events that were positive for C19 marker and negative for CD3.

As in previous studies, the positive/negative limit for FITC fluorescence used for the D8/17 staining was selected for each blood sample to ensure that the irrelevant IgM monoclonal antibody did not represent more than 2% of the positive CD19+ lymphocytes. The D8/17 positive B lymphocytes population was considered to be increased when the proportion of D8/17 within the B lymphocytes CD19+ compartment was above 11% [7].

Streptococcal antibody titers (anti-streptolysin O) were performed using standard semiquantitative procedures. The cutoff for elevated titers, pre-established from the laboratory, was a dilution of 1:200.

### 2.3. Statistical analysis

Non-parametric tests were used for statistical analysis. To compare the medians of percentages of D8/17 in B lymphocytes in different groups of patients the Kruskall-Wallis test was used. The comparison between medians of percentages of D8/17 in patients with ASLO positive and ASLO negative was made using the Mann-Whitney U test. The Spearman non-parametric correlation was used to determine the correlation between D8/17 expression and the Yale-Brown score in OCD patients. A $p$ value lower than 0.05 was considered statistically significant. Statistical calculations were performed using the SPSS for windows, version 10.0.

## 3. Results

### 3.1. Sample characteristics

The patient group had a mean age of 13.1 years (SD = 2.25). Five patients that met PANDAS criteria presented abrupt clinical onset of the symptoms after an otorhinolaryngological infection reported by parents. In all these cases antistreptolysin titers were positive (Table 1). None of the patients had previously been diagnosed with RF or SC, and none had a family history of RF.

Comorbidity was also considered: four patients had two disorders (OCD and TS) and were assigned to one of two groups according to the relative severity of their symptoms. Disruptive behaviour was also diagnosed in one of the patients with OCD. All the OCD patients had a Yale-Brown score between 20 and 40 (mean score of 30 (SD = 5.6); all were moderate or severe. Nine (53%) were severe (score > 30). The TS patients had a mean total Yale Global Tic Severity Scale score of 36.2 (SD = 11.4; range 21–53), and the mean of EAT score in the AN group was 69.8 (SD = 24.18; range 49–97).

Fifteen out of 27 patients (55.5%) were positive for ASLO titers using a semiquantitative procedure. ASLO titers were higher than in similar previous studies in all three groups, but the proportion of positive titers was particularly significant in the OCD and TS groups (58.8% and 60% respectively).

Table 1
Characteristics of subjects whom sera were obtained

| Subject | Age | Gender | Diagnosis | Associated infection | Antistreptolysin titers | D8/17 | IgM |
|---|---|---|---|---|---|---|---|
| 1 | 12 | F | AN | no | 0 | 1.65 | 1.76 |
| 2 | 12 | F | AN | no | 0 | 2.11 | 2.12 |
| 3 | 11 | F | AN | no | 0 | 1.37 | 1.78 |
| 4 | 11 | F | AN | no | 200 | 2.37 | 1.48 |
| 5 | 11 | F | AN | no | 400 | 4.47 | 1.70 |
| 6 | 10 | M | TS | yes | 200 | 4.50 | 1.82 |
| 7 | 11 | M | TS | no | 0 | 2.10 | 1.89 |
| 8 | 12 | M | TS | no | 400 | 2.95 | 1.27 |
| 9 | 14 | F | TS | no | 0 | 3.60 | 1.44 |
| 10 | 11 | M | OCD | no | 800 | 2.21 | 1.76 |
| 11 | 14 | M | OCD | no | 0 | 3.29 | 1.28 |
| 12 | 15 | M | OCD | no | 200 | 6.10 | 1.76 |
| 13 | 12 | M | OCD | yes | 200 | 6.94 | 1.68 |
| 14 | 15 | M | OCD | no | 0 | 1.42 | 1.83 |
| 15 | 15 | F | OCD | no | 400 | 1.89 | 2.07 |
| 16 | 13 | M | OCD | no | 400 | 2.16 | 1.94 |
| 17 | 14 | F | OCD | yes | 400 | 2.11 | 1.88 |
| 18 | 16 | F | OCD | no | 200 | 39.65 | 1.44 |
| 19 | 15 | M | OCD | no | 0 | 2.21 | 2.08 |
| 20 | 13 | F | OCD | yes | 200 | 1.74 | 1.73 |
| 21 | 14 | F | OCD | no | 200 | 3.92 | 1.86 |
| 22 | 16 | F | OCD | no | 200 | 2.53 | 1.88 |
| 23 | 14 | M | OCD | no | 0 | 2.56 | 1.05 |
| 24 | 15 | M | OCD | no | 0 | 5.30 | 2.01 |
| 25 | 15 | M | OCD | no | 0 | 1.70 | 1.45 |
| 26 | 13 | F | OCD | no | 0 | 3.90 | 1.76 |
| 27 | 16 | M | OCD | yes | 200 | 13.10 | 1.82 |

F = female, M = male. AN = Anorexia Nervosa, TS = Tourette syndrome, OCD = Obsessive Compulsive Disorder.

### 3.2. *D8/17 expression in B cells*

As described in the methods section, the cut-off point for the evaluation of the proportion of B lymphocytes positive for the D8/17 marker was set at 11%. The D8/17 positive proportion of B lymphocytes was above 11% in only two out of 17 OCD patients and in none of the TS or AN patients (Table 1). A picture of panels showing the D8/17 expression is represented in Fig. 1.

These two OCD subjects had the highest scores on the Yale-Brown scale and presented the severest symptoms of all the patients. Considering the same parameters as previous studies the expression of D8/17 in B cells was very low: the mean percentage in the entire sample was 4.80, SD 7.36 (minimum 1.37, maximum 39.65) (Table 1). Considered by diagnosis, the mean in the AN group was 2.39, (SD 1.22), in the TS group was 3.43 (SD 0.88) and in the OCD group was 5.91 (SD 9.16). The difference between mean percentages in the three groups was not statistically significant ($p = 0.249$) (Fig. 2).

### 3.3. *Relation between variables*

The correlation coefficient between D8/17 expression and the Yale-Brown score was 0.124, which was not statistically significant (Fig. 3). Comparing D8/17 expression between ASLO positive and ASLO negative subjects (Fig. 4), we found that the mean percentage of B cells expressing D8/17 was not significantly higher ($p = 0.079$, power = 38%) in the ASLO positive group ($n = 15$, mean = 6.4 %, SD = 9.6 %) than in the ASL0 negative group ($n = 12$, mean = 2.7%, SD = 1.2%).

## 4. Discussion

In the present study, an increase in the D8/17 positive B lymphocytes subpopulation was found in only two out of 27 patients. It was not possible to confirm the findings of previous studies, since 92.5% of the patients were negative for the D8/17 marker. Moreover, statistically significant differences in the expression of the D8/17 antigen in the three different groups of patients were not found. The only similarity we found

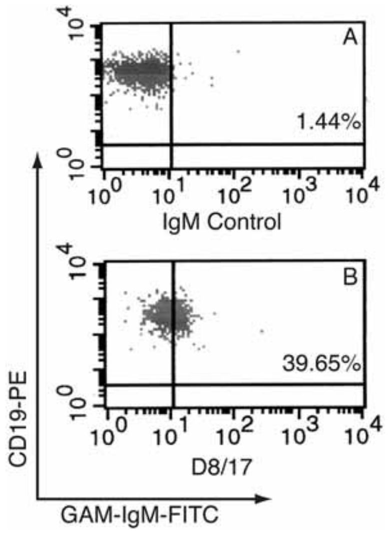

Fig. 1. Example of D8/17 analysis from an OCD positive patient. Each dot represents one cell. Both axes refer to fluorescence intensity reflecting the magnitude of the respective antibody binding (AcMo D8/17, IgM control, Goat AntiMouse(GAM)-IgM, AcMoCD-19) of each cell. The top panel shows the expression in the B cell of an irrelevant monoclonal IgM (IgM control). The bottom panel shows the expression of the D8/17 specific monoclonal antibody. The area of specific binding is defined as the area in which less than 2% of the B cells bind to the irrelevant control IgM.

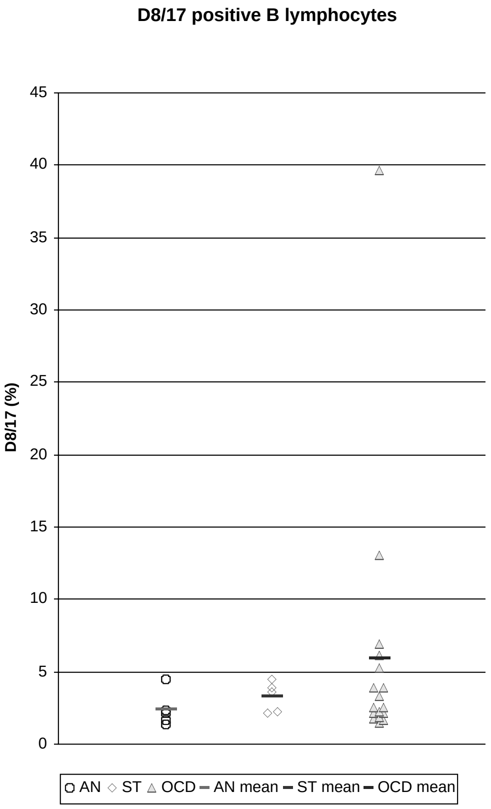

Fig. 2. Percentage expression of D8/17 MoAb in B cells of AN, ST and OCD patients.

in the two D8/17 positive patients was the presence of severe obsessive-compulsive symptomatology. Their Yale Brown scores were 35 and 40, both were resistant to all medication assayed and both have evolved to social and academic maladjustment. Therefore, although the number of patients included in this study is small, it seems that D8/17 positivity may be related to the worst clinical outcome.

One interesting feature of the design of the present study is that we used an irrelevant IgM monoclonal antibody (CD8) to assess D8/17 expression on B lymphocytes. Hoekstra et al. [11] also used this procedure but other studies using cytometry to evaluate D8/17 did not. The use of this irrelevant antibody in our study guarantees that the data obtained are reliable. Although we validated the technique, which is highly reproducible for the monoclonal antibodies used in our laboratory, we were unable to replicate the results with D8/17. This raises doubts about the stability of the antigen, or suggests that the conditions of preservation or purification of the monoclonal antibody may not have been appropriate.

A major limitation of some of the previous positive studies is that a clear characterization of the antigen recognized by D8/17 monoclonal antibody is still lacking. It has been reported that D8/17 binds to myosin and tropomyosin and to streptococcal M proteins [5]. However, it is still not known which (antigen) Ag is recognized at the surface of the B lymphocytes and, therefore, it is not clear how molecular mimicry could act at this level.

In early studies, D8/17 expression was assessed by direct visual evaluation on a fluorescent microscope, a procedure known to have a lower sensitivity and reproducibility, as well as higher subjectivity. Table 2 summarizes the studies of D8/17 conducted to date and shows that confidence in D8/17 as a diagnostic tool is not as high as it was [16]. The recent data published by the group of J.L. Weisz [23] suggest that differences in percentages of D8/17 can be explained by an increased number of CD-19-positive B cells in different populations of patients. Supporting the controversy in the detection of D8/17, Hoekstra et al. [24] have reanalysed their published positive results. They suggested that increased D8/17 expression on B cells can be explained by an overexpression of the constant parts of IgM molecules, indicative of a more general state of immune activation.

Table 2
A comparision of significant studies done with D8/17 since the beginning of its isolation

| AUTHOR | SAMPLE | METHOD | RESULTS (positive for D8/17) |
|---|---|---|---|
| Patarroyo 1979 [27] | 21 RF vs 52 controls | Fluorescence micro | 70% vs 17% |
| Zabriskie 1985 [6] | 24 RF vs 24 controls | Fluorescence micro | 95% vs 21% |
| Khanna 1989 [7] | 84 RF/RHD vs 76 controls | Fluorescence micro<br>Flow cytometry | 98.8%vs 14% |
| Taneja 1989 [28] | 54 RF vs 54 controls | Fluorescence micro | 62.9% vs 12.5% |
| Ganguly 1992 [29] | 90 RF vs 30 controls | Fluorescence micro | 66.4% vs 14% |
| Herdy 1992 [30] | 10 RF vs 8 controls | Fluorescence micro | 38.5% vs 4.6% |
| Murphy 1997 [8] | 31 OCD/ST vs 21controls | Fluorescence micro | 100% vs 5% |
| Sweedo 1997 [4] | 27 PANDAS vs 9 SC vs 24 controls | Fluorescence micro | 85% vs 89% vs 17% |
| Chapman 1998 [31] | 43 OCD/ST vs 31 controls | Fluorescence micro<br>Flow cytometry | 77% vs 13% |
| Niehaus 1999 [32] | 17 Trichotillomania vs 12 OCD vs 22 controls | Fluorescence micro | 58.8% vs 91.6% vs 63.6% |
| Hollander 1999 [14] | 18 autist vs 14 medically ill | Fluorescence micro | 78% vs 21% |
| Murphy 2001 [33] | 32 OCD/CTD vs 12 controls | Flow cytometry | 26% vs 9.1% |
| Hoesktra 2001 [11] | 33 tic disorders vs 20 controls | Flow cytometry | 60.6% vs 5% |
| Eisen 2001 [34] | 29 OCD adults vs 26 controls | Fluorescence micro | 59% vs 42% |
| Sokol 2002 [13] | 16 PANDAS-AN vs 17 psychiatric controls | Flow cytometry | 81% vs 12% |
| Hamilton 2003 [16] | 26SC and 42 OCD or tic disorders vs 19 controls | Florescence micro<br>Flow cytometry | 61.8% vs no data controls |

RF Rheumatic fever; RHD Rheumatic heart disease; OCD Obsessive-compulsive disorder; TS Tourette symdrome; CTD Chronic tic disorder; PANDAS Pediatric Autoimmune Neuropsychiatric Disorder Associated with Streptococcal infection; AN Anorexia Nervosa; SC Sydenham's chorea.

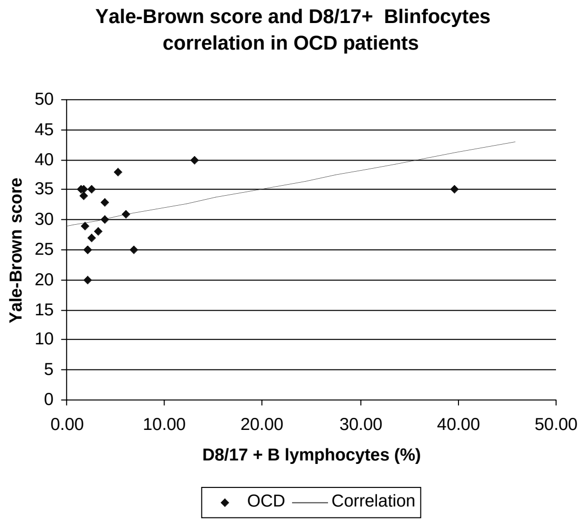

Fig. 3. Correlation between percentage of D8/17 MoAb positive B cells and Yale-Brown Obsesive-Compulsive score for the OCD group.

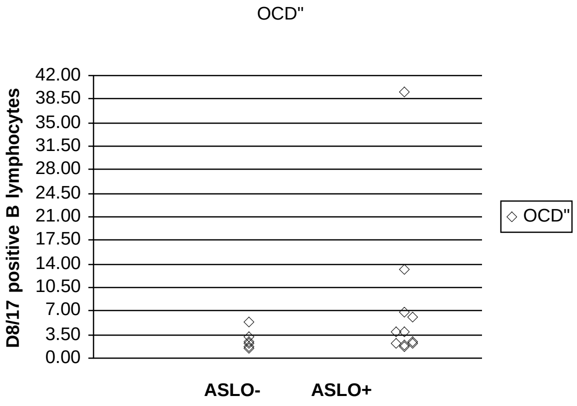

Fig. 4. Percentage expression of D8/17 MoAb in B cells of ASLO negative/ASLO positive patients.

In addition, the study shows differences in percentage levels of D8/17 expression between ASLO positive and ASLO negative patients. However, this observation did not reach statistical significance, which may be due to a type II error (38%). This study also found higher positive ASLO titers (50–60% in the three groups) than other studies had previously found in control populations (20–30%) [25]. These data need to be confirmed with larger samples.

Furthermore, the ethnic or regional origin of patients may be a relevant factor. While almost 100% of rheumatic fever patients in the US have been described to be D8/17 positive, a high expression of D8/17 on B lymphocytes was found in only 66% of RF subjects in India. No data are available in our geographical area [26].

One limitation of the present study was the small number of subjects with prepubertal AN or TS, which makes it hard whether the results will generalize to other patients with the same disorders. The low number of patients included may actually explain some of the negative findings. Another limitation was the lack of a control group from the general population to allow comparison of the percentage of B cells expressing D8/17 antigen in our patients with the percentage in normal children in our area. As previous studies have determined as positive a percentage of B cells expression of D8/17 higher than 11%, in the present study results with a lower percentage were considered as negative. Taking into account that, in the patient group, most of the individuals had a value below 11%, we consider that a negative control group was not necessary to reach our conclusions. However, it also needs to be considered that each specific method produces its own reference values, particularly when applying an isotype control. In this way, it is possible that a control group from our particular area, analysed with this more specific method (isotype control and flow cytometry) would have yielded cut-off values different than the 11% proposed.

The molecular characteristics of D8/17 need to be defined in order to clarify the implication of this antigen in these diseases as different research groups have proposed. Until the positive results are indeed reproduced, the diagnostic value of the antibody remains unclear. It would be important to replicate the studies with the same populations when more purified or modified forms of the monoclonal antibodies become available. Research into the characterization of the antigen recognized by the D8/17 monoclonal antibody is also very important in order to clarify the pathogenic significance of its overexpression, and its hypothetical role as a blood marker for defining homogeneous subgroups of OCD, TS or prepubertal AN patients. The role of autoimmunity in neuropsychiatric disorders is a vast area, but a great deal of work is still to be done if we are to understand its significance. Progress in this field may well lead to the development of new treatments for these diseases, which in many cases are chronic and severe.

## Acknowledgments

The authors thank Dr. Zabriskie (Rockefeller University, NY) for providing the D8/17 monoclonal antibody, and Juanjo Barceló, Yrma Machuca, Angels Hernández, Maria Antònia Romera, Laura Rubio, Josepa Solé for technical assistance and Joan Massana for suggestions related to the project.
